# Post‐bath incontinence (bathwater incontinence) can be managed with behavioral therapy

**DOI:** 10.1002/iju5.12441

**Published:** 2022-03-18

**Authors:** Kumiko Kato, Hiroki Hirabayashi, Aika Matsuyama, Hiroki Sai, Akinobu Ishiyama, Haruka Kurosu, Takashi Kato, Satoshi Inoue, Shoji Suzuki

**Affiliations:** ^1^ Department of Female Urology Japanese Red Cross Aichi Medical Center Nagoya Daiichi Hospital Nagoya Japan; ^2^ Department of Urology Japanese Red Cross Aichi Medical Center Nagoya Daiichi Hospital Nagoya Japan

**Keywords:** bathwater incontinence, behavioral therapy, extraurethral incontinence, post‐bath incontinence, vaginal reflux

## Abstract

**Introduction:**

We encountered six post‐bath incontinence cases caused by bathwater entrapment in the vagina.

**Case presentation:**

The age of onset was distributed from 16 to 78 (average 38) and five out of six patients were parous. Three patients developed post‐bath incontinence immediately after vaginal delivery. One patient developed post‐bath incontinence after beginning to bathe in a reclined position and another after undergoing transvaginal mesh surgery to treat prolapse. All patients showed dribbling incontinence without urgency limited to within 30 min after bathing. Patients were instructed to put a towel between their legs and apply abdominal pressure to evacuate the entrapped water. Additionally, they were advised to squat in the bathtub to prevent water entrapment. This simple behavioral therapy relieved symptoms.

**Conclusion:**

The differential diagnosis of incontinence in women should include entrapped fluid incontinence such as bathwater incontinence, pool water incontinence, and vaginal reflux during micturition.

**Abbreviations & Acronyms**
BMI: body mass index
ICS: International Continence Society
POP: pelvic organ prolapse
SUI: stress urinary incontinence

**Keynote message**

Post‐bath incontinence (bathwater incontinence) can be managed with simple behavioral therapy to evacuate entrapped fluid and/or prevent fluid entrapment. The differential diagnosis of incontinence without urgency in women of any age should include vaginal entrapment such as post‐bath incontinence, pool water incontinence, and urethrovaginal reflux (vaginal reflux).

## Introduction

The ICS states that urinary incontinence should be distinguished from sweating or vaginal discharge.^1^ Vaginal entrapment of bathwater or pool water must be considered as a condition that may be misdiagnosed as urinary incontinence, especially SUI; however, this is not mentioned by the ICS. We present a case series of six women presenting with post‐bath incontinence.

## Case presentation

We retrospectively investigated the medical charts of women who visited our female urology clinic between 2018 and 2021 and found six patients who had experienced post‐bath incontinence. Over that period, 1984 new patients complained of urinary incontinence; thus, post‐bath incontinence comprised 0.3%. Details such as age, parity, BMI, medical history, and method of management were analyzed (Table 1).

**Table 1:** Characteristics of patients who had experienced post‐bath incontinence (bathwater incontinence)

No.	Onset (years)	Parity	BMI	SUI	POP	Medical history	Management
1	16	0	19.2	None	None	Psychosomatic disease	Behavioral therapy
2	27	2	24.1	None	4 years later	Onset after second vaginal delivery	Behavioral therapy
3	29	1	22.9	Simultaneous	28 years later	Onset after first vaginal delivery	Spontaneous relief
4	37	1	22.3	None	None	Onset after first vaginal delivery	Behavioral therapy
5	41	1	28.0	10 years previously	None	Onset after beginning to bathe in a reclined position	Behavioral therapy
6	78	3	22.2	40 years previously	1 year previously	Onset after transvaginal mesh surgery to treat POP	Behavioral therapy

The age of onset was distributed widely from 16 to 78 (average 38.0 ± 21.4) and the average BMI was 23.1 ± 2.9. Three patients developed post‐bath incontinence immediately after vaginal delivery. One patient, 4 years after her first delivery, developed post‐bath incontinence when she moved to an apartment with a full‐sized bath and began bathing in a reclined position. This patient had also experienced pool water incontinence after breaststroke swimming. In the patient with the latest onset (78‐year‐old), post‐bath incontinence began after she underwent transvaginal mesh surgery to treat POP. The only nulliparous patient (16‐year‐old) had no apparent etiology. The majority of patients, at the same time or intertemporally, had SUI (3 patients) and/or POP (3 patients).

All patients showed dribbling incontinence without urgency limited to 10–30 min intervals after bathing. Dribbling usually occurred in an upright position and was worsened by stepping over the edge of the bathtub, bending forward, standing up, sitting down, and walking. In five out of six patients, we diagnosed post‐bath incontinence by observing specific symptoms. In one case, we could gain additional objective evidence by analyzing a sample, which was obtained by asking the patient to evacuate the entrapped fluid by vaginal finger insertion. This sample had the same composition as bathwater: Na 6 mEq/L, K 0 mEq/L, Cl 7 mEq/L, Ca 0.5 mg/dL, UN 0 mg/dL, UA 0.1 mg/dL, Creatinine 0 mg/dL, NAG 0 U/L, protein <5 mg/dL, transparent, specific gravity 1.000, pH 5.0).

**Fig. 1:**
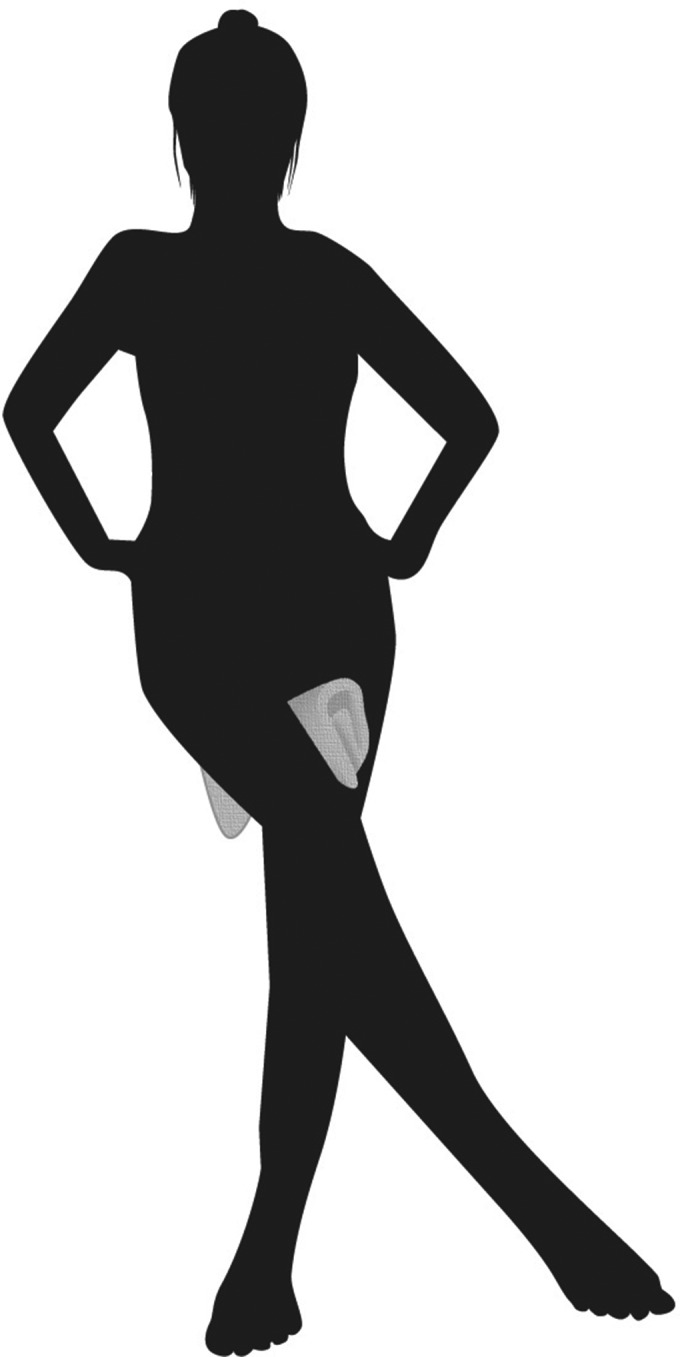
Evacuating entrapped fluid after bathing by putting a towel between the legs and applying abdominal pressure or crossing the legs. This simple behavioral therapy is also useful to manage vaginal reflux and post‐micturition dribble (using toilet paper instead of a towel).

**Fig. 2:**
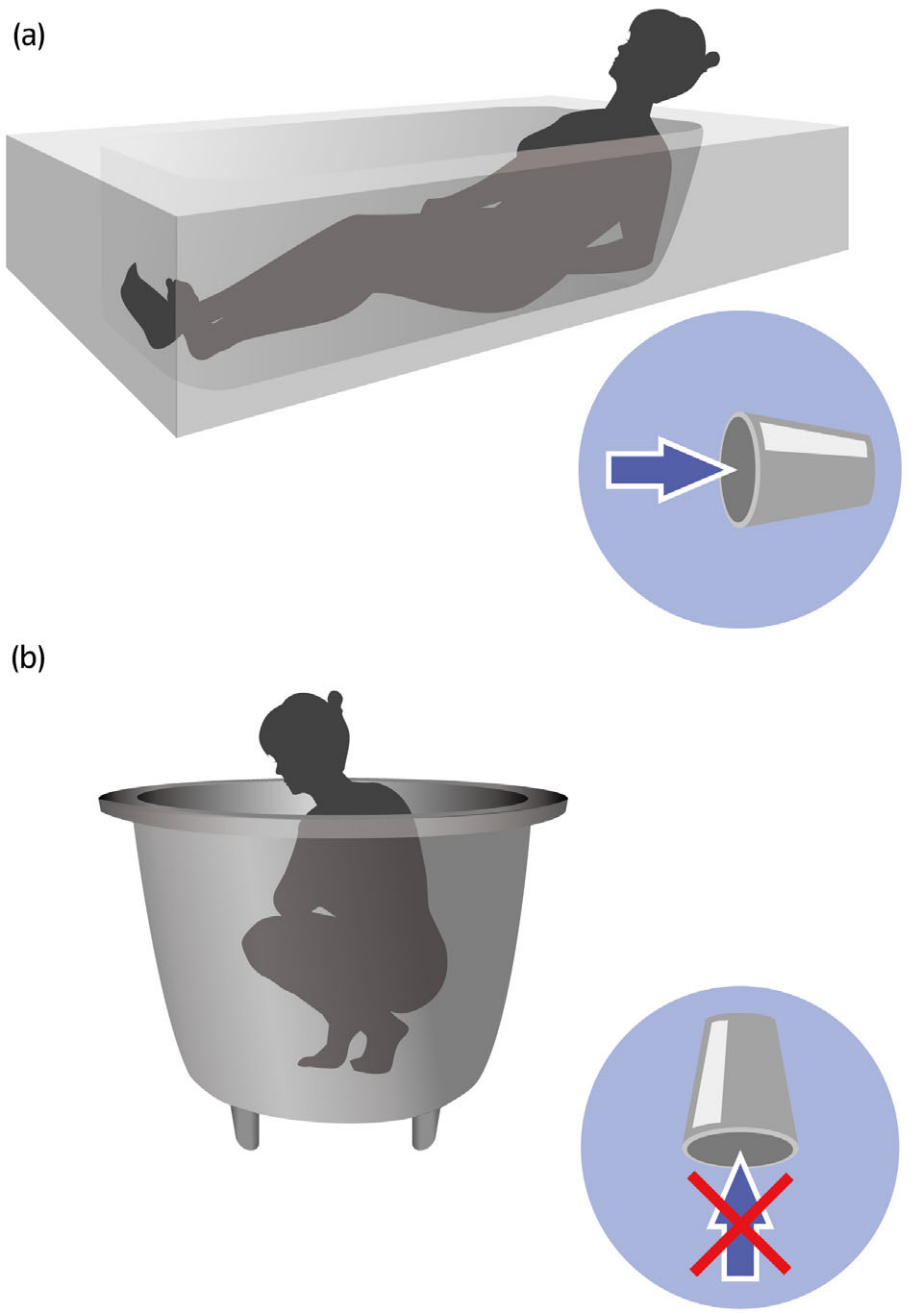
Preventing water entrapment while bathing. (a) Bathwater tends to enter the vagina while a woman reclines in the bathtub. Similar to a cup lying on its side. (b) Bathwater usually does not enter the vagina while a woman squats or kneels in the bathtub. Similar to a cup turned upside down.

The mechanism of post‐bath incontinence was explained, and patients were relieved to learn that this condition is rather common among women and non‐harmful. All patients were instructed to put a towel between their legs and apply abdominal pressure or cross their legs after bathing (Fig. 1). They were also advised to squat or kneel in the bathtub instead of reclining and stretching out their legs (Fig. 2). Such simple behavioral therapy relieved symptoms in five of six patients. The remaining patient had spontaneous relief 2 years after onset and shared this experience during her consultation about POP 30 years later.

## Discussion

We found only one English paper describing post‐bath incontinence.^2^ Psooy *et al*. reported two cases: an 8‐year‐old girl who was advised to shower instead of bathing, and a 39‐year‐old parous woman who, on her own initiative, made a habit of evacuating the entrapped water by inserting a digit in her vagina and pulling laterally. Motavasseli *et al*. reported that vaginal reflux during micturition is an underidentified cause of urinary incontinence in adult women and vaginal finger insertion can be used to manage it as well.^3^ In our experience, many women have feelings of resistance toward vaginal finger insertion. Thus, to evacuate entrapped fluid, it may be a much more acceptable method to put a towel between the legs and apply abdominal pressure or cross the legs after bathing. Additionally, Shigeta *et al*. recommended preventing water entrapment by squatting in the bathtub instead of reclining which is consistent with our study.^4^ Other methods such as pelvic floor muscle training and high‐intensity focused ultrasound have also been tried in Japan.^4^, ^5^

Post‐bath incontinence can be basically diagnosed by confirming characteristic symptoms, i.e., dribbling incontinence without urgency limited to within 30 min after bathing. Psooy *et al*. analyzed the color and pH of the entrapped fluid,^2^ and we carried out further biochemical analysis in one of our cases. We found 1 770 000 results for “bathwater incontinence” and 20 700 000 results for “*oyu‐more*” (bathwater incontinence in Japanese) by doing an Internet search (Google). Therefore, it seems that post‐bath incontinence is not a rare condition and some women have been able to identify and deal with it discretely without consulting medical institutions. Japanese people are known to prefer to bathe rather than to shower, which may explain the difference in the number of results between the English phrase and Japanese phrase.

Post‐bath incontinence often occurs following vaginal delivery and can be accompanied by SUI and POP; thus, one of the main etiologies must be pelvic floor laxity. It may also be associated with the change of Japanese bathing style from a traditional squatting position to a reclining position. Interestingly, one of our patients experienced post‐bath incontinence and a sensation of vaginal openness after POP surgery. It appears that repositioning of the obstructing mass adversely caused these consequences. Both our study, and that of Psooy *et al*.,^2^ included a nulliparous girl. Congenital pelvic floor laxity and/or looseness between anterior and posterior vaginal walls may play a role in developing fluid entrapment. The nulliparous girl in our case series was reluctant to have a vaginal examination. However, authors of Japanese papers^4^, ^5^ noticed pelvic laxity in young nulliparous patients (personal communication).

Although only 0.3% of new patients with incontinence symptoms complained of post‐bath incontinence in our study, some cases may have been overlooked as we did not ask simple questions such as “Do you have incontinence without urgency after bathing?” Half of our patients had complained of post‐bath incontinence to previous doctors, but it was not diagnosed correctly. Even urologists do not always know of the existence of post‐bath incontinence, which may lead to misdiagnosis or delayed diagnosis. The differential diagnosis of incontinence without urgency in women of any age should include vaginal entrapment. It is necessary to check patients' circumstances (when and how leakage occurs) in order to differentiate conditions such as post‐bath incontinence, pool water incontinence, and vaginal reflux. Further epidemiological studies are anticipated.

## Author Contributions

Kumiko Kato: Conceptualization; data curation; formal analysis; writing – original draft. Hiroki Hirabayashi: Supervision; visualization. Aika Matsuyama: Formal analysis. Hiroki Sai: Formal analysis. Akinobu Ishiyama: Formal analysis. Haruka Kurosu: Formal analysis. Takashi Kato: Formal analysis. Satoshi Inoue: Formal analysis. Shoji Suzuki: Supervision.

## Conflict of interest

The authors declare no conflict of interest.

## Approval of the research protocol by an Institutional Reviewer Board

This study was approved by the Ethics Committee of Japanese Red Cross Aichi Medical Center Nagoya Daiichi Hospital (Approval No. 2021–004).

## Informed consent

All informed consent was obtained from the subjects.

## Registry and the Registration No. of the study/trial

Not applicable.
